# APEX2-enhanced electron microscopy distinguishes sigma-1 receptor localization in the nucleoplasmic reticulum

**DOI:** 10.18632/oncotarget.17906

**Published:** 2017-05-16

**Authors:** Timur A. Mavlyutov, Huan Yang, Miles L. Epstein, Arnold E. Ruoho, Jay Yang, Lian-Wang Guo

**Affiliations:** ^1^ Department of Surgery, Wisconsin Institute for Medical Research, University of Wisconsin, Madison, WI 53705, USA; ^2^ Department of Neuroscience, University of Wisconsin, Madison, WI 53706, USA; ^3^ Department of Anesthesiology, Wisconsin Institute for Medical Research, University of Wisconsin, Madison, WI 53705, USA; ^4^ McPherson Eye Research Institute, University of Wisconsin, Madison, WI 53705, USA; ^5^ Department of Surgery, Davis Heart and Lung Research Institute, The Ohio State University, Columbus, OH 43210, USA; ^6^ Department of Physiology and Cell Biology, Davis Heart and Lung Research Institute, The Ohio State University, Columbus, OH 43210, USA

**Keywords:** the sigma-1 receptor, APEX2-enhanced electron microscopy, nucleoplasmic reticulum, nuclear envelope, serial sectioning

## Abstract

The sigma-1 receptor (Sig1R) is an endoplasmic reticulum chaperonin that is attracting tremendous interest as a potential anti-neurodegenerative target. While this membrane protein is known to reside in the inner nuclear envelope (NE) and influences transcription, apparent Sig1R presence in the nucleoplasm is often observed, seemingly contradicting its NE localization. We addressed this confounding issue by applying an antibody-free approach of electron microscopy (EM) to define Sig1R nuclear localization. We expressed APEX2 peroxidase fused to Sig1R-GFP in a Sig1R-null NSC34 neuronal cell line generated with CRISPR-Cas9. APEX2-catalyzed gold/silver precipitation markedly improved EM clarity and confirmed an apparent intra-nuclear presence of Sig1R. However, serial sectioning combined with APEX2-enhanced EM revealed that Sig1R actually resided in the nucleoplasmic reticulum (NR), a specialized nuclear compartment formed via NE invagination into the nucleoplasm. NR cross-sections also indicated Sig1R in ring-shaped NR membranes. Thus, this study distinguishes Sig1R in the NR which could otherwise appear localized in the nucleoplasm if detected with low-resolution methods. Our finding is important for uncovering potential Sig1R regulations in the nucleus.

## INTRODUCTION

The sigma-1 receptor (Sig1R) had long been deemed an orphan receptor or mistaken as an opioid receptor subtype [1]. Later studies revealed Sig1R is unique: 1) It has no homolog in mammalian genomes [2]; 2) its structure is like no other [3]; 3) it represents a new class of signaling modulator [4, 5] whose functions may not be apparent until challenged with stress [1, 6–8].

Sig1R mutations are linked to familial amyotrophic lateral sclerosis (ALS) [9, 10]. Studies with animal models support a protective role of Sig1R in major neurodegenerative diseases, including Parkinson's [11], Alzheimer's [12], ALS [13, 14], as well as retinal degeneration [7, 8, 15–17]. Thus, Sig1R is a probable therapeutic target. There are hundreds of Sig1R-binding compounds [18] with some in clinical use [19, 20] or trials [21, 22]. Moreover, the newly reported Sig1R crystal structure [3] opens a way for optimizing Sig1R drugs for therapeutic development. However, likely due to the lack of homologs, the molecular mechanisms underlying Sig1R functions are poorly understood, impeding Sig1R-targeted therapeutic development.

Sig1R has been characterized as a molecular chaperone [4]. Studies showed that Sig1R resides not only in the endoplasmic reticulum (ER) membrane but also in the nuclear envelope (NE) [4, 23]. Our recent electron microscopic (EM) investigation revealed that Sig1R in retinal neurons is predominantly distributed in the outer and inner NE membranes [24]. Most recently, Tsai et al. reported that Sig1R modulates chromatin remodeling and transcription via the interaction with an inner NE protein [25]. Hence, Sig1R-associated nuclear regulation has emerged as a new frontier of investigation in the elucidation of Sig1R molecular function.

It is also important to note that in many immunocyto/histochemical studies Sig1R has been frequently detected inside the nucleus. For example, in a recent report, immunohistochemistry with postmortem brain tissues of multiple neurodegenerative diseases showed inclusion-like Sig1R immunopositivity inside the nucleus [26]. Given that Sig1R is a membrane protein and there is no prominent membrane structure in the nucleoplasm, the apparent presence of Sig1R deep inside the nucleus seems to contradict its thus-far identified localization in the membranes of NE and ER. Thus an intriguing question arises as to what is the precise Sig1R intra-nuclear localization.

To obtain unambiguous evidence, we applied the APEX2 technology [27, 28]. This recently developed technology proved powerful for ultra-structural studies [29], and indeed substantially improved our ability to distinguish Sig1R subcellular localization. We found that the apparent Sig1R presence inside the nucleus resulted from its localization in the nucleoplasmic reticulum (NR) which are NE invaginations deep into the nucleus, rather than from a presence in the nucleoplasm. This clarification provides new insights potentially important for deciphering the Sig1R function in regulating nuclear activities.

## RESULTS

### Generation of a Sig1R-APEX2 expression cellular model in a Sig1R-null background

**Figure 1:**
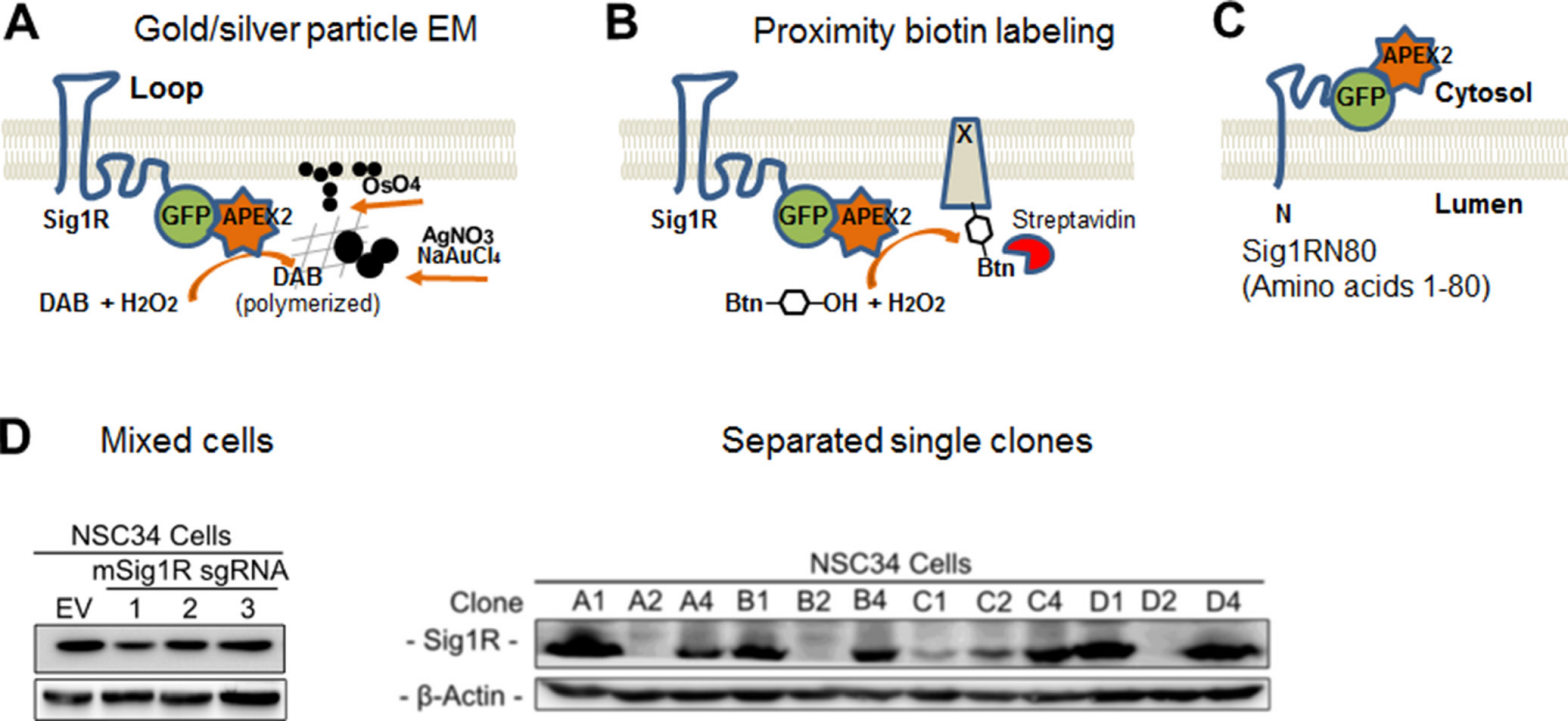
Diagrams of the APEX2 strategy for Sig1R localization and generation of the Sig1R knockout NSC34 cell line with CRISPR-Cas9 (**A**) Diagram of APEX2-enhanced gold particle EM. APEX2 is fused to the C-terminus of Sig1R-GFP and expressed in NSC34 cells. In the presence of H_2_O_2_, APEX2 catalyzes polymerization of DAB which reacts with osmium to produce precipitates that increase EM contrast, or reacts with silver nitrate and gold chloride resulting in easily visualized larger electron-dense aggregates or particles. The Sig1R topology is drawn based on the conventional two-transmembrane model [1]. (**B**) Diagram of APEX2-catalyzed proximity biotin labeling. APEX2 converts biotin-phenol into oxidative radicals which covalently inserts into proteins in close proximity (protein x). Biotinylated proteins can be visualized by fluorescently labeled streptavidin revealing Sig1R localization. (**C**) Diagram showing GFP-APEX2 fused to the C-terminus of Sig1RN80 (amino acids 1-80), which is proposed to be on the cytosolic side of the ER membrane [1, 3]. (**D**) Identification of effective Sig1R sgRNAs and selection of Sig1R KO NSC34 cell single clones. Cas9-positive cells were enriched with 1 μg/ml puromycin for 7 days. Cells expressing sgRNA No.1 were serially diluted and expanded for single clone selection. EV: empty vector.

APEX is a peroxidase originally engineered for use in high resolution electron microscopy (EM) [28]. Typically, APEX is fused to a protein of interest and expressed in cells. Fixed cells are incubated with diaminobenzidine (DAB) and H_2_O_2_. Catalyzed by APEX, polymerized DAB deposits recruit electron-dense osmium thus producing local EM contrast (diagramed in Figure 1A). Alternatively, the APEX fusion protein can also be expressed for proximity-dependent biotin labeling of neighboring proteins [27] followed by visualization via fluorescence microscopy (Figure 1B).

To study subcellular localization of Sig1R via APEX-enhanced EM and APEX-catalyzed proximity biotin labeling, we chose the second-generation enzyme (APEX2) developed in the Ting lab [27]. APEX2 is small (27 kDa), monomeric, and free of disulfide bonds and thus stable in various intracellular environments [27]. Using a Sig1R-GFP vector that we previously developed, we created constructs of Sig1R-GFP-APEX2 to express the full-length Sig1R protein fused to APEX2 (Figure 1A), and Sig1RN80-GFP-APEX2 with an N-terminal Sig1R fragment fused to APEX2 (Sig1RN80, i.e., amino acids 1–80, Figure 1C). GFP was included to monitor fusion protein expression and as an alternative indicator of Sig1R subcellular localization.

**Figure 2:**
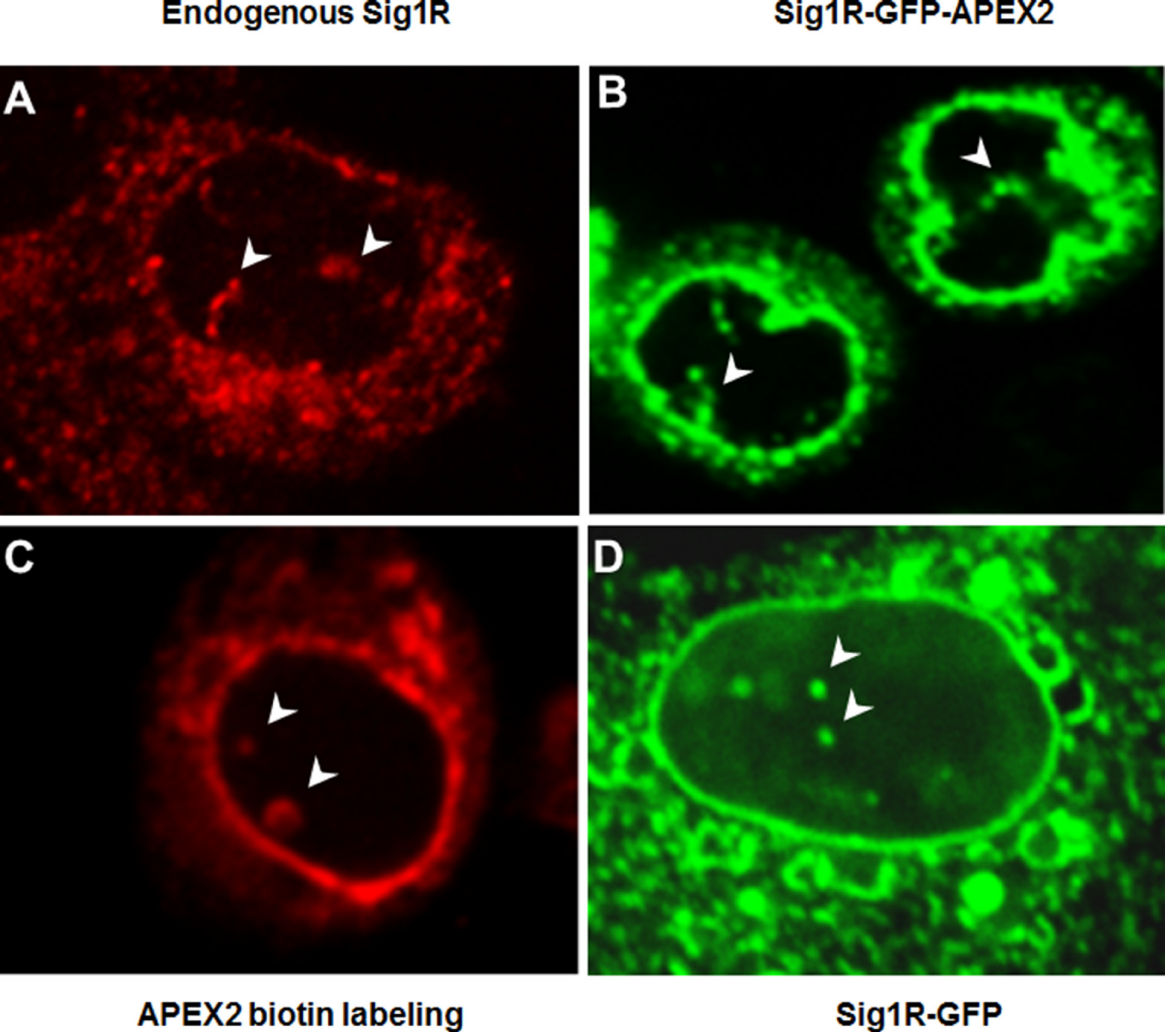
Different fluorescence microscopy methods identify Sig1R inside the nucleus (**A**) Endogenous Sig1R was detected in NSC34 cells by immunocytochemistry using an in-house produced Sig1R antibody [40] with confirmed minimal non-specific labeling [7, 24]. (**B**) Sig1R-GFP-APEX2 fusion protein was expressed in a Sig1RKO NSC34 cell line (see Figure 1D). GFP fluorescence imaging indicates antibody-free Sig1R subcellular localization. (**C**) Sig1R-GFP-APEX2 fusion protein was expressed in Sig1RKO NSC34 cells followed by APEX2-catalyzed biotin labeling of proteins proximal to APEX2 (and hence Sig1R), whose localization was visualized via Cy3-labeled streptavidin. (**D**) Sig1R-GFP fusion protein was expressed in a Sig1RKO ARPE19 cell line (generated using the same method as in Figure 1D) and visualized by fluorescence microscopy. In all images, arrowheads indicate Sig1R localization inside the nucleus. Note the Sig1R-positive tubular nuclear structures within the nucleus.

In order to express the Sig1R-GFP-APEX2 fusion protein in a homogeneous cellular background, we generated a Sig1R-null NSC34 neuronal cell line using the CRISPR-Cas9 technology. To knock out Sig1R from NSC34 cells, we chose a genome-editing approach using a lentiviral vector expressing Cas9 and a Sig1R sgRNA. Single clones of Sig1R knockouts were selected, and verified by Western blotting for depletion of the Sig1R protein (Figure 1D). In subsequent experiments, we used single clone D2 with clear loss of Sig1R immunoreactivity. A Sig1R-null ARPE19 cell line (Figure 2) was also generated using this method.

### APEX2 proximity biotin labeling indicates Sig1R inside the NSC34 cell nucleus

To reproduce the previously reported immuno-detection of Sig1R in the nucleus [26, 30], we immunostained *endogenous* Sig1R in wild type NSC34 cells. As shown in Figure 2A, Sig1R-positive staining is clearly visible in the nucleus. To exclude the possibility that the Sig1R immunoreactivity inside the nucleus results from a non-specific antibody effect, we next determined Sig1R subcellular localization via fluorescence imaging of the Sig1R-GFP fusion protein without the need to use an antibody. We transfected NSC34 cells with the Sig1R-GFP-APEX2 fusion construct and then performed fluorescence microscopy. Like the endogenous Sig1R protein detected by immunostaining, Sig1R-GFP-APEX2 visualized by GFP fluorescence was found in a tubular intra-nuclear structure (Figure 2B). This experiment also indicates that the distribution of overexpressed Sig1R protein in subcellular compartments was similar to that of endogenous Sig1R.

To further confirm this observation, we next applied APEX2 proximity biotin labeling [27], a technique which illuminates Sig1R localization via a mechanism distinct from the first two approaches. The NSC34 cells transfected with the Sig1R-GFP-APEX2 fusion construct were exposed to biotin-phenol and then H_2_O_2_. The basic principle is that catalyzed by the APEX2 peroxidase activity, the proteins next to APEX2 (and hence Sig1R) are labeled by biotin which can then be visualized using fluorescently labeled streptavidin (see Figure 1B). As shown in Supplementary Figure 1, fluorescence from the Cy3 label in streptavidin (red) appeared only in the Sig1R-GFP-APEX2 transfected cells exposed to both biotin-phenol and H_2_O_2_, illustrating the superior specificity of this method. Neither of the controls, i.e., un-transfected cells, or transfected cells treated with only H_2_O_2_ or biotin-phenol, displayed detectable red fluorescence. Moreover, red fluorescence and green fluorescence (from Sig1R-GFP-APEX2) overlapped well (Supplementary Figure 1), validating the method of APEX2 proximity biotin labeling for subcellular localization of Sig1R. Importantly, using this method we again observed the Sig1R presence in the nucleus (Figure 2C). In order to examine whether the observed nuclear distribution of Sig1R is NSC34 cell type-dependent, we transfected a Sig1R-null ARPE19 retinal pigment epithelium (RPE) cell line with a Sig1R-GFP (no APEX2) fusion construct. While Sig1R localized by GFP fluorescence was found predominantly in the NE and the ER network, its intra-nuclear distribution was also observed (Figure 2D). Taken together, the results from three different fluorescence imaging approaches and two distinct cell lines consistently revealed Sig1R distribution in the nucleus.

### APEX2-enhanced gold-particle EM shows Sig1R residing in the ER at the ER/mitochondria contacts with unprecedented clarity

Fluorescence microscopy is typically limited by two prominent shortcomings. First, the resolution is too low to provide detailed information of precise subcellular protein localization. Second, the multi-plane imaging depth may produce false-positive Sig1R signal in the nucleus. We therefore opted to use an APEX2-based strategy, which has been recently advanced by Ting's group and proven powerful for enhancing EM [29]. As diagramed in Figure 1A, in the presence of H_2_O_2_, APEX2 (fused to Sig1R) catalyzes DAB polymerization and subsequent osmium precipitation and locally produces EM contrast [27]. We further improved this antibody-free EM method by applying silver/gold precipitation (Figure 1A and Supplementary Figure 2), a technique we have used in traditional EM [24]. We found that APEX2-catalzed silver/gold precipitation generated sizable EM dots that render Sig1R localization highly visible.

**Figure 3:**
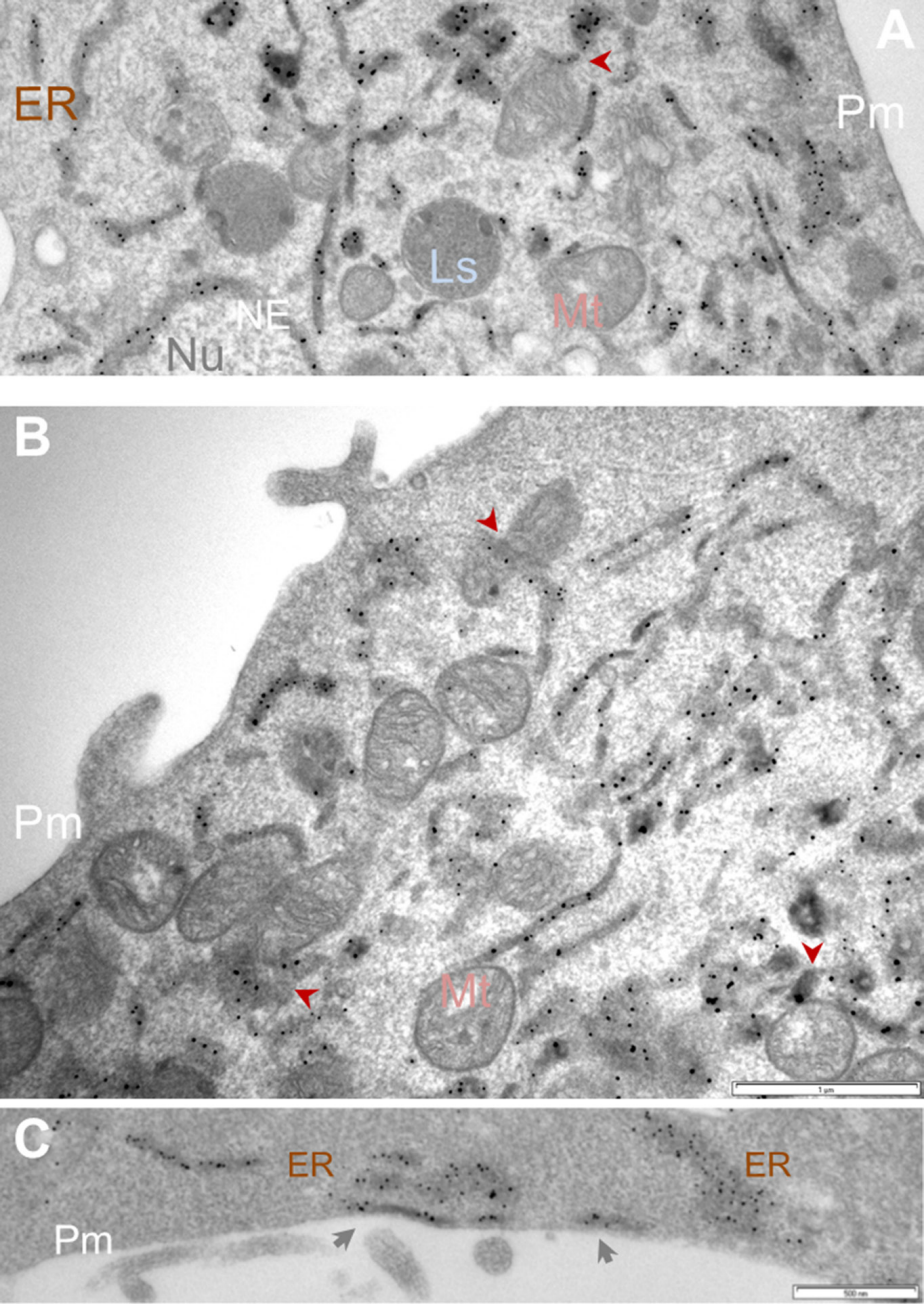
APEX2-enhanced gold particle EM detects Sig1R localization in the ER network and at the ER/mitochondria contacts Sig1R-GFP-APEX2 fusion protein was expressed in Sig1RKO NSC34 cells, followed by cell fixation, sectioning, and APEX2-enhanced gold-particle EM, as described in Methods. Shown in (**A** and **B)** are images from different cells. Arrowheads point to examples of Sig1R localization at the ER sites that juxtapose with mitochondria which are identified by the presence of characteristic membranous cristae. Image (**C)** highlights Sig1R localization in subsurface cisternae (arrows). ER: endoplasmic reticulum; Ls: lysosome; Mt: mitochondria; NE: nuclear envelope; Nu: nucleus; Pm: plasma membrane. Scale bar: 1 μm in A and B; 0.5 μm in C.

Using this method, we observed Sig1R-indicative EM dots predominantly in the ER and the NE (Figure 3), an expected result based on previous reports [4, 23, 24]. We also found Sig1R-indicative dots in the ER membranes that juxtaposed with mitochondria readily identified by their clear membranous cristae. These ER/mitochondria contacts presumably represent mitochondria-associated ER membrane (MAM), a site where Sig1R is known to reside and play an important role in mitochondrial homeostasis [4]. We were not able to detect Sig1R in the plasma membrane in our experimental setting, although Sig1R has been suggested to relocate to the plasma membrane under certain circumstances [31]. Instead, we observed Sig1R-indicative APEX/EM dots at the ER sites that juxtapose with the plasma membrane (Figure 3C), namely, subsurface cisternae, consistent with our previous immuno-EM results visualizing endogenous Sig1R in neurons [24, 32, 33]. Taken together, the known Sig1R localizations in the NE, ER and subsurface cisternae validate the effectiveness of the APEX2-based strategy for ultra-structural study of Sig1R. In addition, our use of this APEX2/gold-particle EM approach has produced unprecedented clarity of the Sig1R subcellular localization and permitted its visualization even at ER/mitochondria contacts.

### APEX2-enhanced gold-particle EM reveals Sig1R in the NSC34 cell nucleus

**Figure 4:**
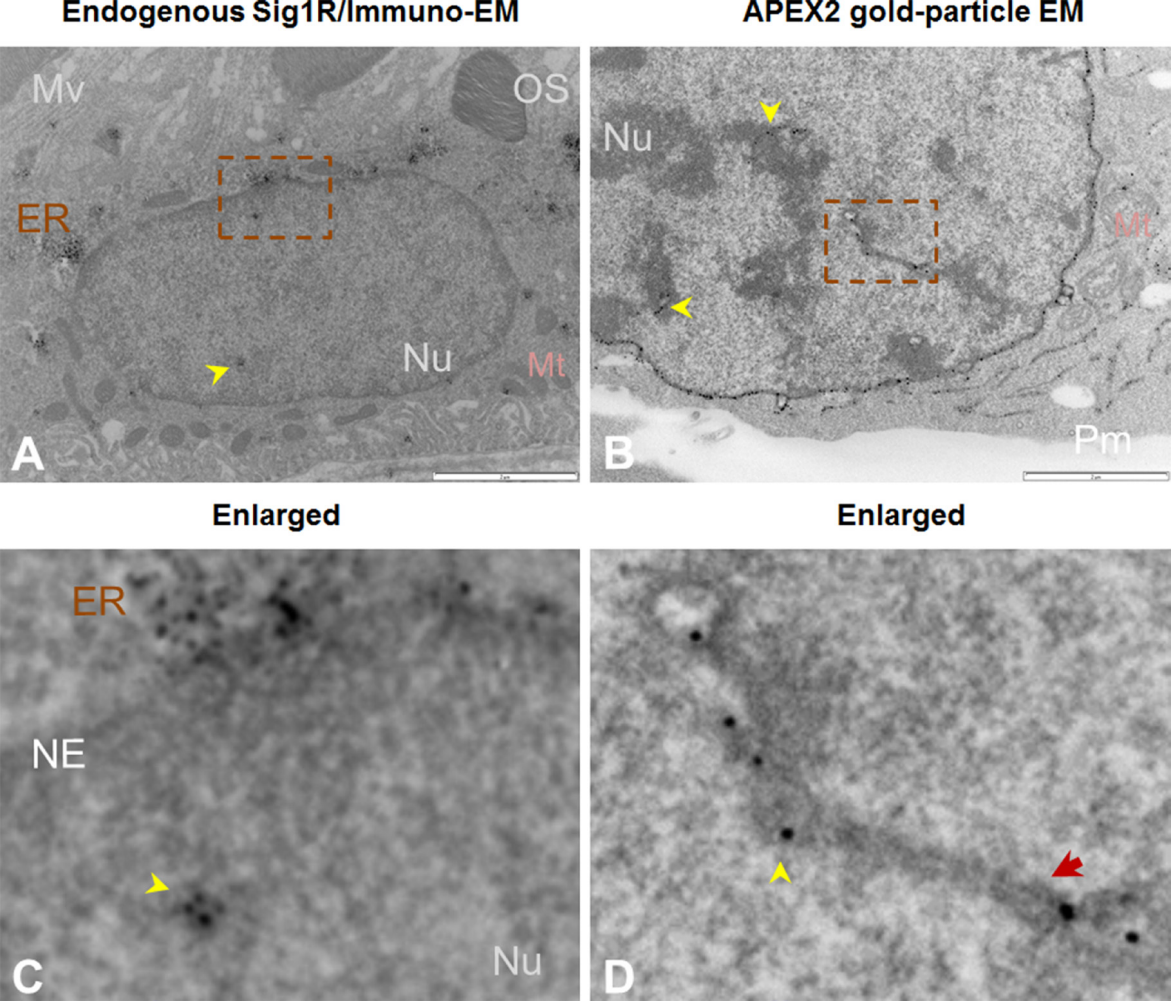
Both immuno-EM and antibody-free APEX2-enhanced gold-particle EM visualize Sig1R localization inside the nucleus (**A**) Immuno-EM showing Sig1R-positive dots (arrowheads) inside the RPE cell nucleus. EM was performed with cross-sections of albino mouse eyes. Sig1R was also detected in the ER. (**B**) APEX2-enhanced gold-particle EM showing Sig1R localization deep inside the NSC34 cell nucleus (arrowheads). Scale bar: 1 μm. (**C** and **D**) Enlarged images of the boxed areas in A and B, respectively. The red arrow in D points to a tunnel-like membrane structure. ER: endoplasmic reticulum; Mt: mitochondria; Mv: microvilli; NE: nuclear envelope; Nu: nucleus; OS: photoreceptor outer segment; Pm: plasma membrane.

Taking advantage of this APEX/EM approach, we re-examined the presence of Sig1R in the nucleus observed via fluorescence microscopy. We first conducted a traditional immuno-EM experiment using an in-house produced Sig1R antibody [7] that we previously used [24], to detect Sig1R in the RPE where no EM localization had been reported. To minimize heavy background imposed by melanin in pigmented mice, we used eye sections from albino mice. The EM images in Figure 4A and 4C) show Sig1R-positive dots inside the RPE cell nucleus and in the peri-nuclear ER. This is the first ultra-structural documentation of *endogenous* Sig1R localization in RPE cells. However, specificity of antibody-based method is subject to the specificity of the antibody used. To eliminate this concern, we resorted to the antibody-free APEX-based strategy. Interestingly, using the APEX2-enhanced gold-particle EM protocol, we were able to clearly visualize Sig1R-indicative, electron-dense EM dots in the nucleus of transfected NSC34 cells (Figure 4B and 4D). Combined, the foregoing results obtained with a variety of approaches demonstrate that the detection of Sig1R inside the nucleus is Sig1R-specific rather than artifactual.

### Serial sectioning combined with APEX2-enhanced gold-particle EM distinguishes Sig1R in the nucleoplasmic reticulum membranes of NSC34 cells

**Figure 5:**
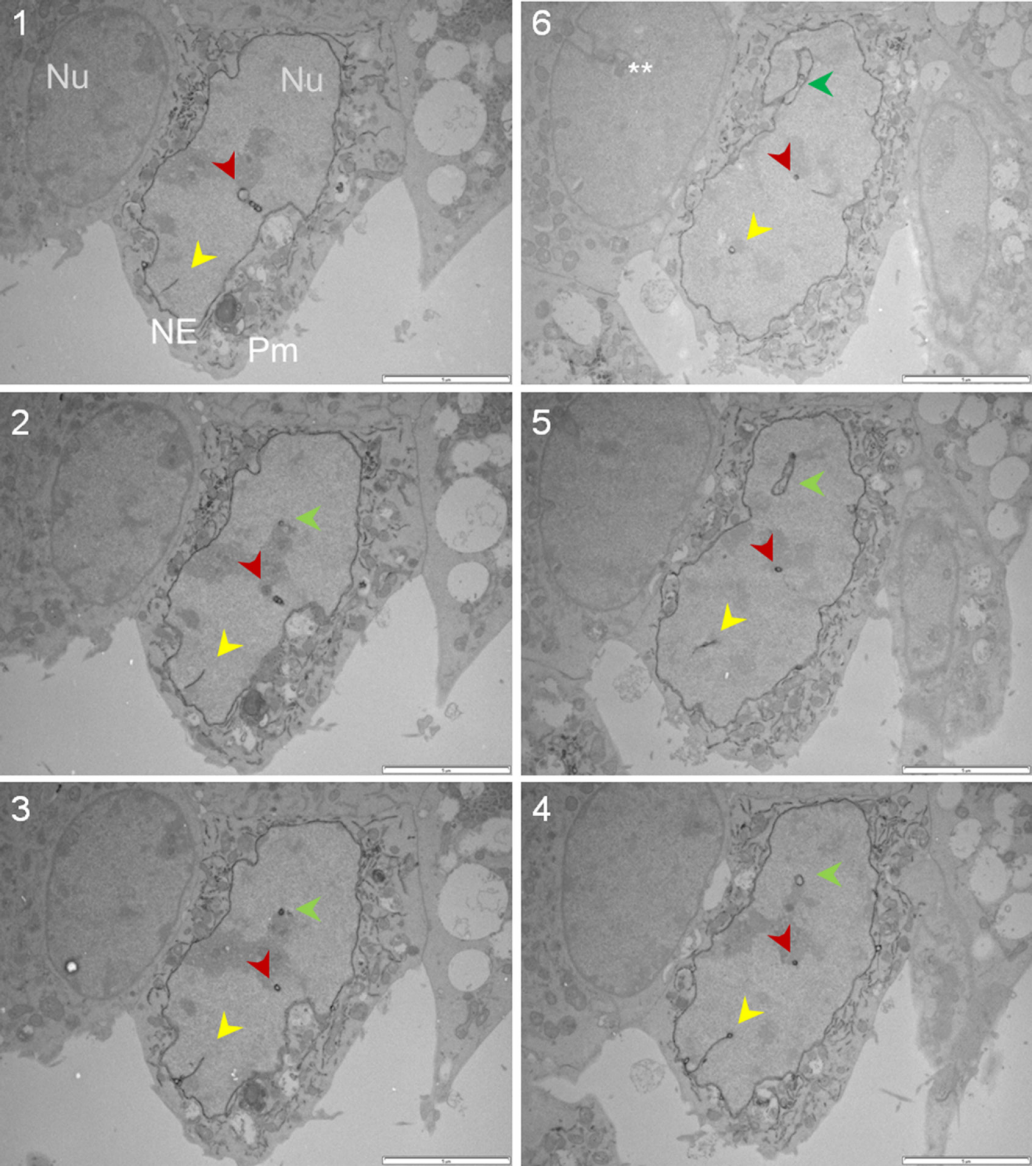
Sig1R is detected in the NR via serial sectioning followed by APEX2-enhanced gold-particle EM The Sig1R-GFP-APEX2 fusion protein was expressed in Sig1RKO NSC34 cells, followed by cell fixation, serial sectioning, and APEX2-enhanced gold-particle EM, as described in Methods. The sequence of images is indicated by numbers. Each panel shows a transfected cell (middle) and an un-transfected cell (left); this un-transfected internal control showing no Sig1R-indicative dots supports the specificity of the APEX2 gold-particle EM method. The yellow, red, and green arrowheads highlight gradual changes of three NR structures. Note one NR tubular structure eventually connect to the NE (green arrowhead, **Image #6**). **NE invagination also occurred in un-transfected cells. NE: nuclear envelope; Nu: nucleus; Pm: plasma membrane. Scale bar: 2 μm.

**Figure 6:**
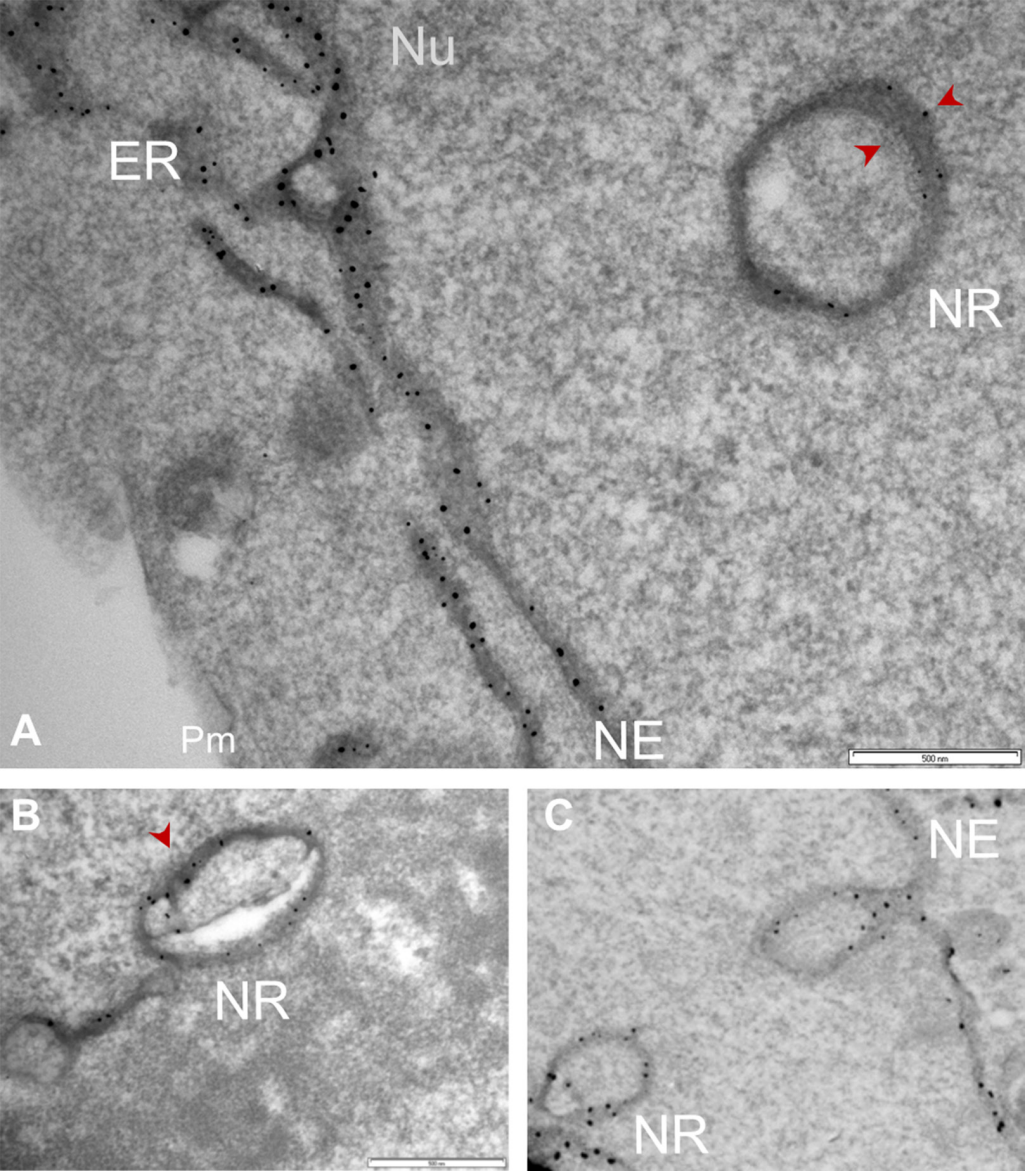
Sig1R localization in the NR membrane cross-sections detected by APEX2-enhanced gold-particle EM Full-length Sig1R-GFP-APEX2 (in **A** and **B**) or Sig1RN80-GFP-APEX2 (**C**) fusion protein was expressed in Sig1RKO NSC34 cells, followed by cell fixation, sectioning, and APEX2-enhanced gold-particle EM, as described in Methods. Images shown in A-C reveal Sig1R-indicative dots in the membrane of NR cross-sections. Sig1R localization is also shown in the NE membrane. Note the NE invagination in (C) forming a NR structure with an opening toward the cytosol. ER: endoplasmic reticulum; NE: nuclear envelope; Nu: nucleus; Pm: plasma membrane. Scale bar: 0.5 μm.

Up to this point, the presence of Sig1R in the nucleus appeared real. However, we observed that Sig1R-indicative dots were distributed along a trace of “tunnel-like” structure in the nucleus (Figure 4D). This pattern of Sig1R distribution prompted us to examine the possibility that Sig1R actually resides in the invaginated NE. We used a serial sectioning strategy to de-convolute the three-dimensional Sig1R distribution inside the nuclear sphere. Interestingly, as the sequential images unroll (Figure 5 and Supplementary Figure 3), the Sig1R-indicative EM signal inside the nucleus first appears as a dot (see Supplementary Figure 3) and then extends into a tube-like structure and finally connects to the NE as an integral membrane structure (Figure 5). As the images continue, the tubular structure “dissolves” and changes into a small ring (Figures 5 and Supplementary Figure 3). Similar transformations were found in different intra-nuclear areas. These data strongly suggested that some pockets of Sig1R-residing NE membranes invaginate to produce tunnel-like structures, namely, nucleoplasmic reticulum (NR) that extends deeply into the spherical geometry of the nucleus [34]. In support of this assertion, reexamination of the fluorescence images in Figure 2 also reveals tunnel-like intra-nuclear Sig1R-positive traces connecting to the NE, in un-transfected (i.e. endogenous Sig1R distribution) (Figure 2A) as well as in transfected cells (Figure 2B). Importantly, we also found Sig1R-indicative EM dots in some membranous ring structures inside the nucleus (Figure 6). These membrane structures are consistent with cross-sections of NR, and appear to contain both inner and outer membranes (Figure 6A). Particularly revealing, Figure 6C captures the anatomy of an invaginating NE tunnel with an opening toward the cytosol, presumably an image of NR structure in the process of being formed. In summary, these data together indicate that although on a single plane of the nucleus Sig1R may appear to reside in the nucleoplasm, in-depth analysis of EM images obtained with various sectioning angles reveals its likely localization to be in the NR.

## DISCUSSION

We have produced compelling evidence to solve a long-held puzzle with regards to intra-nuclear Sig1R localization. We addressed it by coupling the APEX technology [28] to the gold/silver precipitation method to improve EM resolution and by applying a serial sectioning strategy to de-convolute the three-dimensional nuclear distribution of Sig1R. Following the expression of Sig1R-GFP-APEX2 in a Sig1R-null neuronal cell line, we attained high-quality EM imaging that clearly indicates Sig1R in the nucleus. Further analysis of serial (and cross) sections revealed that while appearing intra-nuclear, Sig1R actually resides in the NR that projects from the NE deep into the nuclear sphere. This elucidation could not have been achieved with traditional antibody-based EM or fluorescence microscopy.

This finding is of timely importance in light of rapidly growing interest in studying Sig1R in disease and its treatment [5]. Sig1R as a unique molecular chaperone [4] regulates diverse cellular activities [5], such as ER stress response, channel activity, GPCR signaling, Ca^2+^ homeostasis, and autophagy etc. Of particular interest, Tsai et al recently identified a Sig1R/emerin interaction, presumably in the inner NE, that regulates gene expression via chromatin remodeling [25]. Miki et al recently reported Sig1R-positive intra-nuclear immunostaining in post-mortem brain samples [26] as well as in cultured HeLa cells [30], an observation which was interpreted as Sig1R protein aggregates entrapped in the nucleus. This is a reasonable idea considering that a Sig1R mutation (E102A) found in familial ALS predisposes Sig1R to aggregation [10]. However, it remains unknown what type of membrane structure in the nucleoplasm Sig1R resides in and how such an aggregate might translocate into the nucleus.

The simplest interpretation of our APEX/EM data is that the Sig1R protein detected inside the nucleus resides in the NR membrane and not in the nucleoplasm. The serial-section EM images clearly reveal the presence of Sig1R along the NR tubular structure. Moreover, NR cross-sections exhibit its double-membrane formation wherein the Sig1R-indicative EM dots are clearly visible. In fact, we previously observed *endogenous* Sig1R (via immuno-EM) in the invaginated NE membrane of ganglion cells in the mouse retina [24]. We did not observe here a Sig1R presence in the nucleoplasm. Sig1R is a membrane-residing protein [3], but there are no obvious membranous structures found in the nucleoplasm. Thus, it is reasonable to infer that other than the NR there is no appropriate membrane environment in the nucleoplasm to maintain a native Sig1R protein structure. Furthermore, there is no nuclear-localizing sequence found in the Sig1R protein [2], although we cannot rule out the possibility of Sig1R entering the nucleus by “piggybacking” onto other proteins such as TDP-43 [26]. Based on our high-resolution EM data we propose that the previously observed apparent Sig1R presence inside the nucleus most likely reflects a NR localization that could not be discerned by traditional low-resolution microscopy.

Our finding of Sig1R in the NR has important implications. Growing evidence indicates that the NE is not simply a physical barrier separating the nucleoplasm from the cytosol [34]. The NR network or NE invaginations are a specialized and dynamic nuclear compartment [34]. NR expansion has been linked to aging, Alzheimer's disease [35], and cancers. While still not well understood, their potential roles range from transcriptional regulation, Ca^2+^ signaling, lipid metabolism to stress response [34]. While a Sig1R modulation of transcription was recently reported [25], the other aforementioned roles ascribed to the NR are reminiscent of well-documented functions of Sig1R [5]. A fascinating fact is that the NR network provides deep-reaching sites within the nucleus that are capable of “conventional” NE functions [34]. These NE sites are otherwise peripheral and inaccessible to nuclear activities, such as transcription and nucleophagy [36]. Moreover, the NR network effectively multiplies the inner NE membrane area, and hence provides better opportunities for inner NE proteins (e.g., Sig1R [24] and emerin [25]) to interact with nucleoplasmic machineries. In this regard, the NR is an ideal platform for possible Sig1R-associated nuclear regulation. On the other hand, since Sig1R resides in the NE, it is also possible that its distribution in the NR is merely a passive consequence of NE invagination. A relatively low density of Sig1R detected in the NR may reflect its differential distribution in the NR and NE membranes. Alternatively, this may result from relatively sparse NR in the cells that were cultured under normal conditions; there is evidence for that NR occurrence is significantly enhanced under stress or disease conditions [35]. To delineate these questions, more detailed investigation is required to explore Sig1R functions in the NR, and may open a new research avenue.

Another interesting question potentially addressable by the high-resolution EM technique pertains to the topology of Sig1R and localization of the Sig1R C-terminal chaperonin domain [4]. There are two major hypotheses: Model 1- Previous hydrophobicity analysis of the Sig1R sequence and experimental evidence support a model whereby Sig1R contains two transmembrane helices (linked with a cytosolic loop) and a hydrophobic C-terminal domain, i.e., the chaperonin domain [4]; both the C-terminal domain and the N-terminal domain (~ 8 amino acid) reside in the ER lumen (diagramed in Figure 1A). This has been the prevailing working model used by most researchers [1]. Model 2- Based on the Sig1R crystal structure that also specifies an N-terminus in the ER lumen followed by a single transmembrane helix with the loop region and the C-terminal domain residing on the cytosolic side of the ER membrane [3]. To gain information of the Sig1R topology, we generated a second construct (see Figure 1C) by fusing GFP-APEX2 to the C-terminus of the Sig1R N-terminal fragment (Sig1RN80, amino acids 1-80) that includes the N-terminal transmembrane helix and the loop domain (~33**−**80). As APEX2 is at the C-terminus of the fusion protein, the APEX2-catalyzed EM dots could reveal on which side of the ER membrane the Sig1R C-terminus resides. Our preliminary results indicate that when APEX2 was fused to the C-terminus of the full-length Sig1R (i.e., Sig1R-GFP-APEX2), the EM dots were localized predominantly on the luminal side of the ER or NE membranes (Figure 3; Figure 6A; Supplementary Figure 4). Distinct from this result, when fused to the Sig1R cytosolic loop (i.e., Sig1RN80-GFP-APEX2, see Figure 1C), a good portion of the EM dots were seen on the cytosolic or nucleoplasmic side of the membrane (Supplementary Figure 4, yellow arrows). These results suggest that placing APEX2 to the C-terminus of the full-length Sig1R protein or that of Sig1RN80 led to different localizations of the fusion protein C-terminus. The cytosolic/nucleoplasmic localization of Sig1RN80-GFP-APEX2 EM dots is consistent with both models but luminal localization of full-length Sig1R-GFP-APEX2 implies the presence of a second transmembrane domain (i.e. Model 1 with two transmembrane helices), or alternatively, a mixed insertion topology of Model 2 with a single transmembrane domain.

However, we are not able to draw a conclusion due to several caveats. First, we were not able to define the Sig1R N-terminal localization because fusing GFP-APEX2 to the Sig1R N-terminus would likely disrupt the ER-targeting of the N-terminal domain which contains an ER locating sequence [4]. Second, the arm length of the GFP-APEX2 fusion protein linker may give rise to flexibility leading to mislocalization of the C-terminal end. This may account for the observation that some of the Sig1RN80-GFP-APEX2 EM dots were on the luminal side, although this localization could have resulted also from sectioning artefact. On the other hand, flexibility of the full-length Sig1R-GFP-APEX2 could have been effectively restrained by the putative second transmembrane helix [1] (Figure 1A), as evidenced by its EM dots predominantly localized on one (luminal) side of the membrane. In addition, since Sig1RN80 is a half molecule, it is not known whether the expressed Sig1RN80-GFP-APEX2 fusion protein assumes the native Sig1R orientation. While final resolution of the Sig1R insertion topology must await more definitive evidence from future research, the high-resolution EM approach described in the present work is likely to provide the answer.

New information obtained in this study was made possible by technical improvement, i.e., the APEX2-coupled EM [27] further enhanced with our silver/gold precipitation protocol [24]. While APEX2 proved powerful to produce high-quality organelle-specific EM images [27, 29], we tailored this technology to meet our specific need of discerning Sig1R nuclear localization. Compared to the APEX/EM using osmium (Supplementary Figure 2A), the Sig1R-indicative EM dots produced by APEX oxidation together with silver/gold precipitation (Supplementary Figure 2B) generated unprecedented clarity of Sig1R localization in the NR, NE, and ER, all three sharing the same contiguous membrane and lumen [34]. A prominent feature of the APEX approach is the omission of antibody, an approach circumventing common issues such as insufficient antibody quality and nonspecific background. As such, this antibody-free EM method of Sig1R-GFP-APEX2-catalyzed gold/silver particle formation confers unique strengths for applications where immuno-EM is problematic. A good example is RPE, which is notoriously challenging for immuno-EM because of the heavy background associated with melanin pigments [37]. A possible future application is to express Sig1R-GFP-APEX2 in the RPE via subretinal delivery of a viral vector, for ultra-structural study of Sig1R distribution in RPE cells.

## MATERIALS AND METHODS

### Animals

All animal procedures conform to the NIH Guide for the Care and Use of Laboratory Animals. The animal protocol (M02102) was approved by the Institutional Animal Care and Use Committee at the University of Wisconsin-Madison. All surgeries were performed under isoflurane anesthesia (flow rate 2 ml/min). Albino mice (stock# 000058) were purchased from the Jackson Laboratory (Bar Harbor, ME), and maintained on a 4% fat diet (8604 M/R, Harkland Teklad, Madison, WI) and subjected to standard light/dark cycles (12 h/12 h). Animals were euthanized in a chamber gradually filled with CO_2_.

### Generation of APEX2-expressing constructs for Sig1R subcellular localization

To generate the Sig1R-GFP-APEX2 fusion construct, the DNA sequence between the *EcoRI* and *BamHI* digestion sites flanking the human Sig1R gene (*SIGMAR1*) was amplified by PCR from pCI-neo- Sig1R-3XFLAG. The DNA sequence between *BamHI* and *NotI* digestion sites flanking GFP-APEX2 was amplified from the plasmid pcDNA3 Connexin43-GFP-APEX2 (Addgene, cat#49385). The amplified Sig1R and GFP-APEX2 DNA fragments were then ligated into an empty vector of pEGFP-N1 (Clontech) cleaved by *EcoRI* and *NotI*. To generate the Sig1RN80-GFP-APEX2 construct for expressing a partial Sig1R molecule that includes amino acids 1-80 (Sig1RN80), we substituted the full-length Sig1R gene with the Sig1RN80 DNA fragment that was PCR-amplified. The Sig1R-GFP fusion construct was generated by inserting the full length Sig1R sequence into *EcoRI* and *BamHI* digested pEGFP-N1.

### Cell culture

The NSC34 mouse motoneuron cell line was purchased from Cellutions Biosystems (Westbury, NY). Human ARPE19 cells were purchased from American type culture collection (ATCC, Manassas, VA). Both cell types were grown in 50%/50% DMEM/F12 supplemented with 10% fetal bovine serum (FBS) and 1 × penicillin/streptomycin (final 100 μg/ml) at 37°C in 5% CO_2_.

### Knockout of Sig1R in NSC34 and ARPE19 cells via CRISPR/Cas9

To generate Sig1R knockout NSC34 and ARPE19 cell lines, a CRISPR/Cas9 genome-editing approach was used, as described in our previous report [38] with minor modifications. Briefly, 3 CRISPR single-guide RNAs (sgRNAs) targeting the Sig1R gene (*Sigmar1*) were chosen. Targeted sequences for the NSC34 mouse cell line: 5′-TGATCCAGGCCGCCTGGTTG-3′, 5′-CGTGGGCCGCGGGACGGCGG-3′ and 5′-GCAGC TTGCTCGACAGTATG-3′. Targeted sequences for the ARPE19 human cell line: 5′-GGCCTTCTCTCG TCTGATCG-3′, 5′-TGACCCAGGTCGTCTGGCTC-3′, and 5′-GTGGGCCGTGGGCCGGCGGT-3′. Cloning of sgRNAs into lentiCRISPR v2 and lentivirus production were performed as reported [39]. Lentivirus was packaged as we previously reported [38]. The cells were transduced with lentivirus for 3 days, then treated with 1μg/ml puromycin for 1 week. Single clones were picked after serial dilution and expanded.

### Western blotting to verify knockout of the Sig1R protein

Immunoblotting was performed as described in our previous publications [38]. Briefly, protein concentrations of cell lysates were determined using a Bio-Rad DC™ Protein Assay kit. Proteins of 50μg from each sample were separated by 12% SDS-PAGE and transferred to a PVDF membrane (Millipore). After blocking, the PVDF membrane was incubated with a mouse anti-Sig1R antibody (sc-13705, Santa Cruz, 1:100 dilution) or a mouse anti-β-actin antibody (A2228, Sigma-Aldrich,1:5000 dilution). After incubation with an HRP-conjugated secondary antibody (goat anti-mouse, Jackson ImmunoResearch Inc., 1:5000 dilution), specific protein bands on the blot were visualized by applying enhanced chemiluminescence reagents according to the manufacturer's instructions (Pierce, Rockford, IL) and then recorded with a LAS-4000 Mini imager (GE, Piscataway, NJ).

### Immunocytochemistry for Sig1R subcellular localization

Coverslips (Fisher #12-545-82) were pre-cleaned overnight in Aqua Regia, rinsed three times with double distilled H_2_O and stored in 100% ethanol. On the day of seeding, coverslips were placed into each well of a 24-well cell culture plate (Costar #3524), dried and coated with 0.01% poly-L-ornithine (Sigma-Aldrich #5666) for 30 min, rinsed 3× with double distilled H_2_O, 10 min each time, and then aspirated and dried under UV illumination.

NSC34 cells were split to 1,000 cells/coverslip and grown in a 37°C incubator with 5% CO_2_ for 72 h in DMEM supplemented with 10% (vol/vol) of cosmic calf serum (HyClone #SH30087-03) and a 1× non-essential amino acid solution (Sigma-Aldrich #M7145) to reach a well-spread morphology and 70–80% confluence. Cells were briefly rinsed (30s) twice in 1× DPBS with CaCl_2_ and MgCl_2_ (100 μg/ml) and fixed for 15 min with a sterile-filtered 1× DPBS solution containing 3.7% paraformaldehyde (PFA) and 0.02% picric acid, pH 7.4. After fixation, cells were rinsed 3× with 1× PBS, quenched with 100 mM glycine in 1× PBS for 3 min, rinsed 3× again with 1× PBS each for 3min, and then permeabilized with 0.1% Triton X-100 for 3 min. Blocking reagent (10% normal goat serum, Biomeda # ES1028) with 0.1% Triton X-100 was applied for 15 min. Cells were incubated with the in-house produced rabbit polyclonal Sig1R antibody [40] for 1h and rinsed 3× with 1× PBS containing 0.1% Triton X-100. The secondary antibody, Alexa594-conjugated goat-anti-rabbit Fab (ThermoFisher #A-11072) was incubated with the cells for 1h. The cells were rinsed 3′ with 1× PBS (0.1% Triton), twice with 1× PBS only, stained with 300 nM DAPI for 5 min, rinsed 3× again with 1× PBS each for 1min, aspirated, and then embedded into Prolong Gold mounting media (ThermoFisher #P36970), dried overnight, and fixed to the glass slide using clear nail polish (Electron Microscopy Sciences # 72180). Images were taken under a 100× objective, with a Nikon Eclipse Ti inverted microscope, or an Andor Revolution XD confocal microscopy system.

### APEX2-catalyzed proximity-dependent biotin labeling for Sig1R subcellular localization

NSC34 cells were grown as described above and transfected with the Sig1R-GFP-APEX2 or Sig1RN80-GFP-APEX2 fusion construct using Lipofectamine2000 (ThermoFisher). After transfection for 24 h, the culture was changed to normal growth medium and cells were incubated with 500 μM Biotin-Phenol (AdipoGen Life Sciences) for 30 min and then with 1 mM H_2_O_2_ for 1min only. The reaction was stopped with a quencher (10mM Sodium Azide, 10 mM Sodium Ascorbate, 5 mM Trolox in 1x DPBS). Cells were washed immediately, fixed in 4% PFA for 20 min, washed again, and residual aldehyde residues were quenched in 20 mM Glycine for 10 min. After another wash, cells were permeabilized with 0.1% TritonX-100 for 20 min and then incubated with Cy3-congugated streptavidin (Biolegend #405215, 500 ng/ml) for 1h for detection of proteins that were in close proximity to Sig1R and hence biotinylated via the APEX2 peroxidase activity. Cells were post-stained with DAPI and mounted to the glass slide in mounting media (Plolong Diamond, ThermoFisher). Images were taken as described above.

### Immuno-electron microscopy (Immuno-EM)

We performed immuno-EM experiments following our published methods [24]. Albino mice (2 months old) were intracardially perfused with 4% PFA and 0.2% glutaraldehyde in 0.1 M phosphate buffer. Eyeballs were dissected and post-fixed in the same fixative overnight. Retinas were dissected and 60 μm thick sections were cut using a Leica VT 100S vibratome. Sections were quenched in 1% sodium borohydrate for 30 min, rinsed with PBS and permeabilized in 0.05% Triton X-100 for 15 min, and then blocked in normal goat serum for 1 h. Sections were incubated with primary anti-sigma-1 receptor antibody (1/150 dilution in PBS) for 48 h at 4 °C. Immunostaining was further revealed with ABC peroxidase kit (Vector Laboratories, Burlingame, CA, USA) and a mixture of 0.02% diaminobenzidine and 0.01% H_2_O_2_ in 50 mM Tris, pH 7.6 for 10 min. The sections were then rinsed and post-fixed with 2% glutaraldehyde for 30 min followed by washing 3× in 100 mM Tris-Maleic Acid. Electron-dense polymer of diaminobenzidine was further intensified by a mixture of 0.52% hexamethyltetramine, 0.04% silver nitrate, and 0.04% sodium tetraborate all in 100 mM Tris-Maleic Acid buffer pH 7.4 for 10 min at 60 °C in the dark. Then the sections were rinsed in nanopure H_2_O and 0.01 M PBS and placed in 0.05% solution of gold chloride for 5 min. To wash away unbound silver particles samples were first treated with 3% sodium thiosulfate for 2 min, and then washed 3× in water. The samples were then post-fixed with 1.5% osmium tetroxide for 1 h, rinsed and stained en block with 1% uranyl acetate and dehydrated in graded series of ethanol, washed twice with propylene oxide (5 min each time). Samples were further infiltrated in Epon resin/propylene oxide (1:1 ratio), and then in pure Epon, and finally polymerized between two teflon coated glass slides. Thin sections of 70 nm were cut using a Leica EM UC7 ultramicrotome, counterstained in 8% uranyl acetate and viewed and imaged with Phillips CM120 STEM electron microscope.

### APEX2-enhanced silver/gold precipitation for EM and serial sectioning for three-dimensional Sig1R nuclear localization

NSC34 cells (Sig1R knockout) were seeded on Poly-D-Lysine coated coverslips in DMEM supplemented with 10% FBS. When cells reached 70% confluence, they were transfected by Lipofectamine 2000 with the Sig1R-GFP-APEX2 construct in Opti-Mem. After 24-h transfection cells were fixed in 2% Glutaraldehyde for 20 min, rinsed 3´, residual aldehydes were quenched in 20 mM Glycine for 10 min. Cells were further rinsed and diaminobenzidine (0.5 mg/ml) and 0.01% H_2_O_2_ were added for 7 minutes to develop dense precipitate. Cells were further postfixed in 2.5% glutaraldehyde for 20 min, washed 3× in 100 mM Tris-maleic acid pH7.4. Electron-dense polymer of diaminobenzidine was further intensified by a mixture of 2.6% hexamethyltetramine, 0.2% silver nitrate, and 0.2% sodium tetraborate all in 100 mM Tris- maleic acid buffer for 10 min at 60°C in the dark. The sections were rinsed in nanopure H_2_O and 0.01M PBS and placed in 0.05% solution of gold chloride for 5 min. To wash away unbound silver particles, samples were first treated with 3% sodium thiosulfate for 2 min, and then washed 3× in water. The samples were post-fixed with 1% osmium tetroxide/1% potassium ferrocyanide for 1 h, rinsed and stained en bloc with 1% uranyl acetate, followed by dehydration in graded series of ethanol and washed twice with propylene oxide (5 min each time). Samples were further infiltrated in Epon resin/propylene oxide (1:1 ratio), and then in pure Epon, and finally polymerized in 60°C oven overnight. Then glass coverslip was dissolved away by placing samples in hydrofluoric acid for 3 hours. Serial thin sections 60 nm thin were cut using a Leica EM UC7 ultra-microtome, counterstained in 8% uranyl acetate, and viewed and sequentially imaged with Phillips CM120 STEM electron microscope.

## CONCLUSIONS

Using diverse approaches capitalizing on the antibody-free APEX2 technology, we addressed a long-standing knowledge gap regarding subcellular distribution of Sig1R in the nucleus. Our results distinguish that Sig1R resides in the NR membrane rather than in the nucleoplasm of NSC34 neuronal cells. The demonstration of Sig1R in the NR is the first step but further research would advance our understanding of the role of Sig1R in regulations of nuclear activities and in NR biology.

## SUPPLEMENTARY MATERIALS FIGURES



## Abbreviations

Sig1R: sigma-1 receptor
ER: endoplasmic reticulum
NR: nucleoplasmic reticulum
NE: nuclear envelope
EM: electron microscopy
