# Supplementary material for: APEX2-enhanced electron microscopy distinguishes sigma-1 receptor localization in the nucleoplasmic reticulum
Source: Oncotarget. 2017 May 16;8(31):51317–30. doi: 10.18632/oncotarget.17906 (PMC5584251; doi:10.18632/oncotarget.17906)
Supplement: Supplementary file 1 [file oncotarget-08-51317-s001.pdf]

APEX2-enhanced electron microscopy distinguishes sigma-1 receptor localization in the nucleoplasmic reticulum

SUPPLEMENTARY MATERIALS

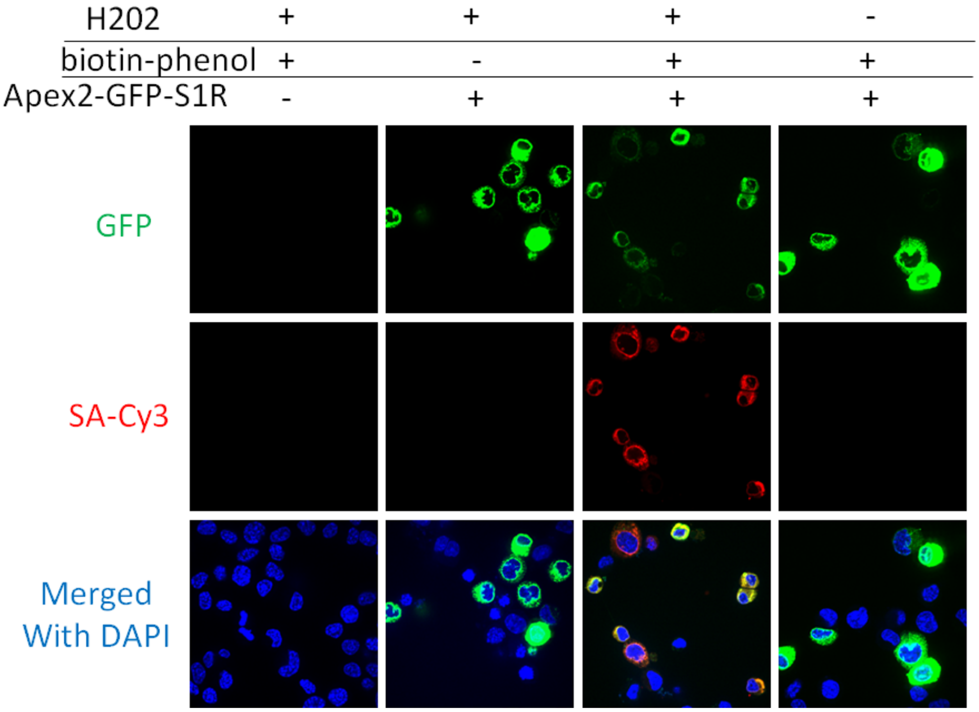

Supplementary Figure 1: APEX2-catalyzed proximity biotin labeling for specific detection of Sig1R subcellular localization. Sig1R-GFP-APEX2 was expressed in Sig1RKO NSC34 cells, and proximity biotin labeling and fluorescence microscopy was performed as described in Methods. SA-Cy3 signal was observed only when all reagents were present. Green: GFP fluorescence from the Sig1R-GFP-APEX2 fusion protein. Red: fluorescence from the Cy3 label on streptavidin (SA). Blue: DAPI.

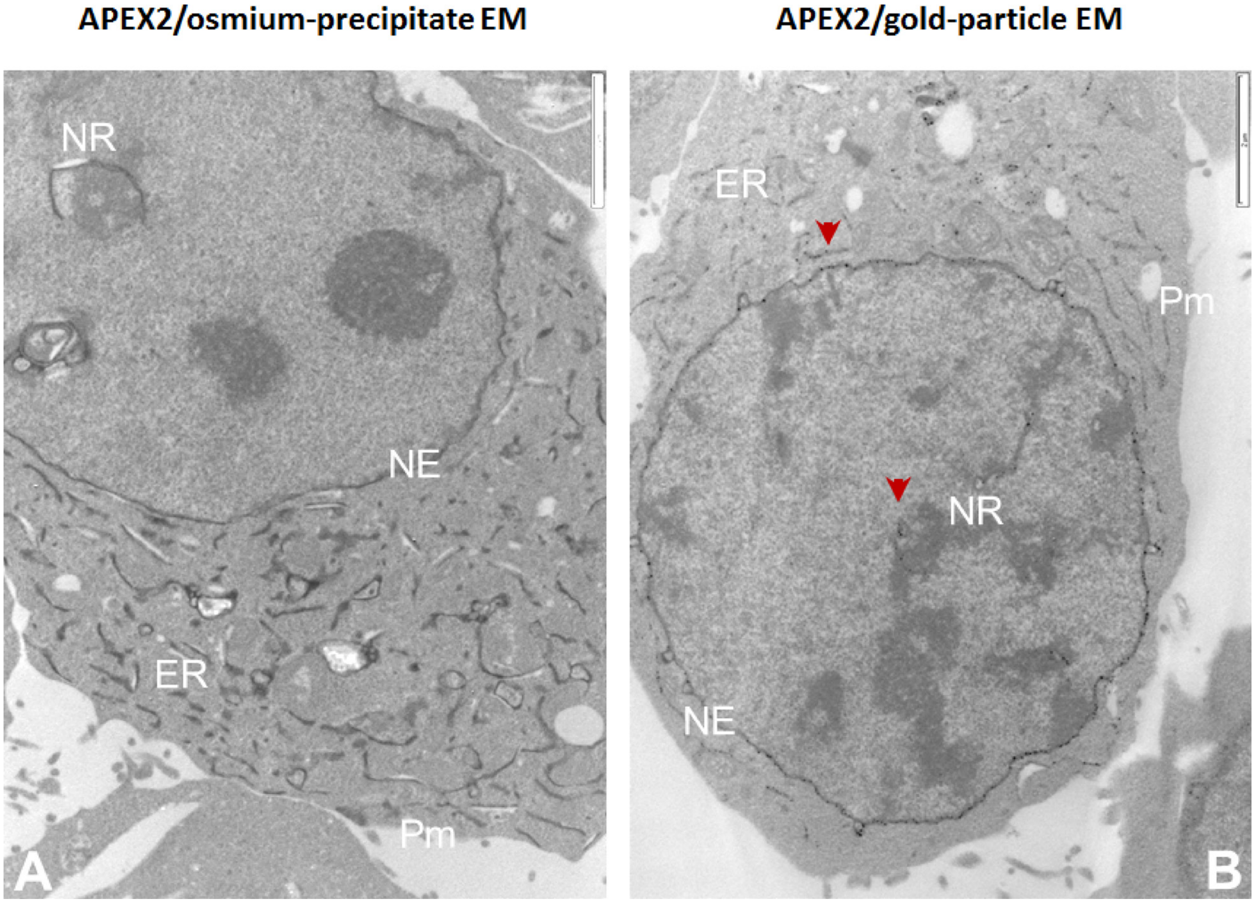

Supplementary Figure 2: Comparison of APEX2-catalyzed osmium precipitation EM with APEX2-enhanced gold-particle EM for Sig1R detection. Sig1R-GFP-APEX2 fusion protein was expressed in Sig1RKO NSC34 cells, followed by cell fixation, sectioning, and APEX2-enhanced osmium precipitation (**A**) or gold-particle EM (**B**), as described in Methods. ER: endoplasmic reticulum; NE: nuclear envelope; Pm: plasma membrane. Scale bar: 2 µm.

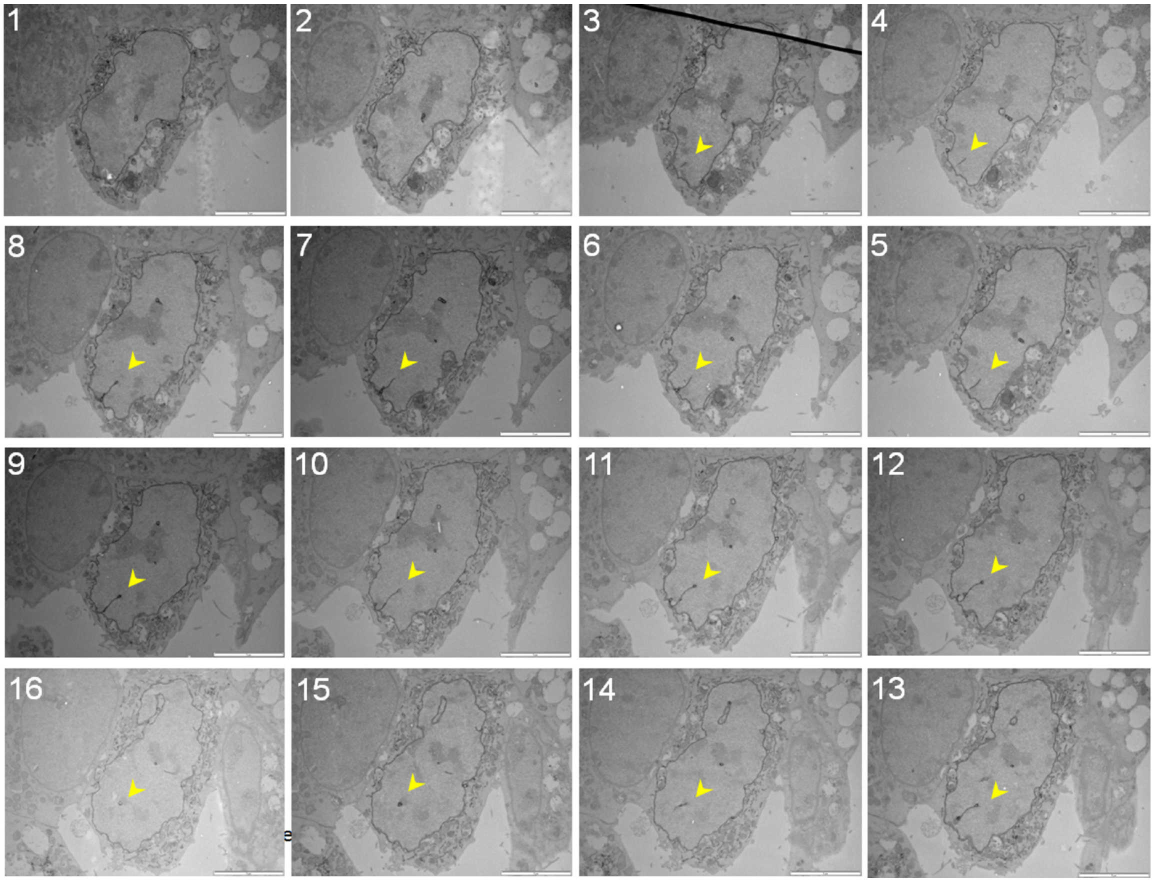

Supplementary Figure 3: Full display of sequential images of APEX2-enhanced gold-particle EM. from serial sections Sig1R-GFP-APEX2 fusion protein was expressed in Sig1RKO NSC34 cells, followed by cell fixation, serial sectioning, and APEX2-enhanced gold-particle EM, as described in Methods. It is the same experiment as in Figure 5. Arrow head points to a NR structure. Scale bar: 2 μm.

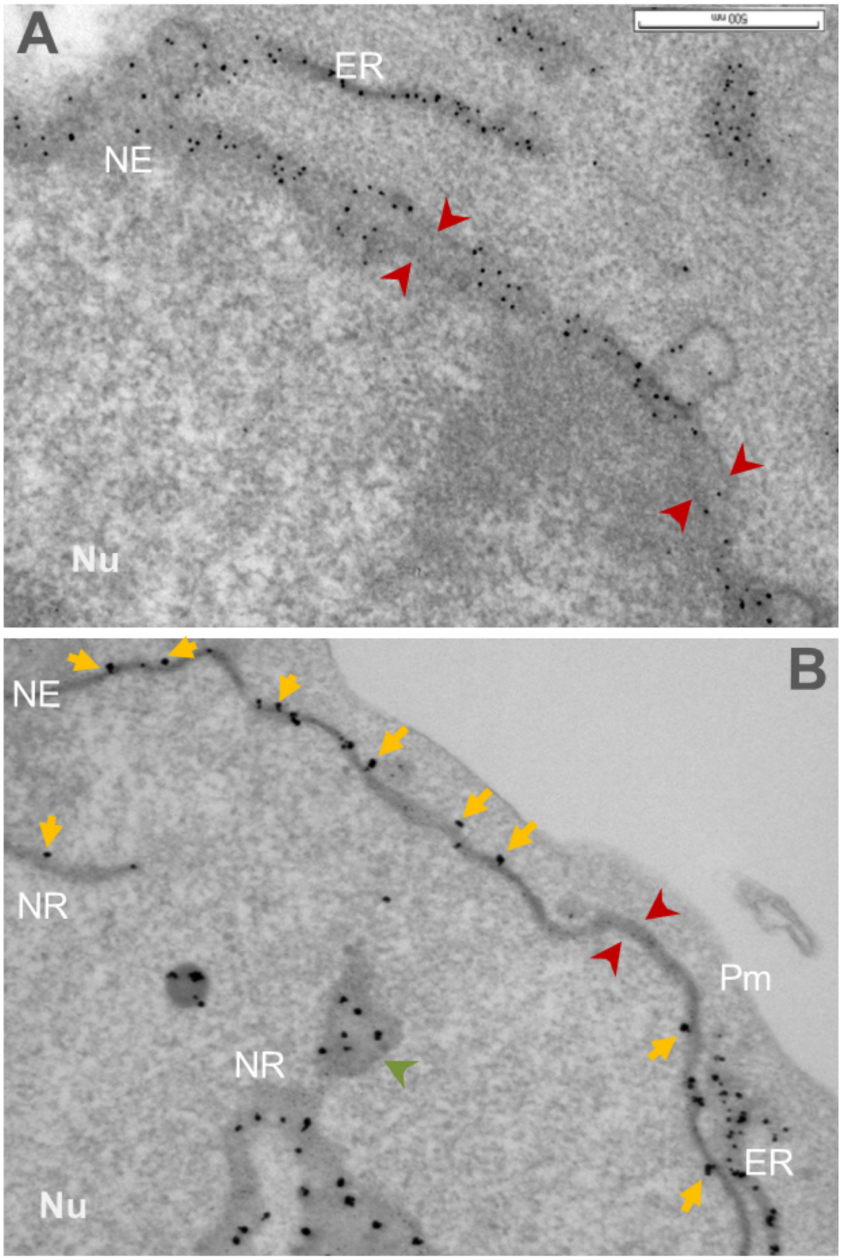

Supplementary Figure 4: Images of APEX2-enhanced gold-particle EM dot distribution in cells expressing Sig1R-GFP-APEX2 and Sig1RN80-GFP-APEX2. Full-length Sig1R-GFP-APEX2 or Sig1RN80-GFP-APEX2 fusion protein was expressed in Sig1RKO NSC34 cells, followed by cell fixation, sectioning, and APEX2-enhanced gold-particle EM, as described in Methods. Shown in (**A** and **B)** are images from Sig1R-GFP-APEX2 and Sig1RN80-GFP-APEX2, respectively. Pairs of arrowheads highlight the double-membrane NE structure. Yellow arrows indicate EM dots oriented toward the cytosolic side or nucleoplasmic side. NE: nuclear envelope; NR: nucleoplasmic reticulum; Nu: nucleus; Pm: plasma membrane. The green arrowhead marks a NR structure that was presumably flat-cut. Scale bar: 0.5 µm.
